# Self-interestedness in Research Collaboration and its Association with Career Stage and Nature of Collaboration: A Survey of Zimbabwean Researchers

**DOI:** 10.1177/15562646231192808

**Published:** 2023-08-01

**Authors:** Similo Ngwenya, Nelius Boshoff

**Affiliations:** 1 Centre for Research on Evaluation, Science and Technology (CREST) and DSI-NRF Centre of Excellence in Scientometrics and Science, Technology and Innovation Policy (SciSTIP), 26697 Stellenbosch University, Matieland, South Africa

**Keywords:** Africa, career stage, collaboration, collaborators, gender, self-interestedness, unethical behaviour

## Abstract

During collaboration in research, it may happen that some researchers become involved in behaviours that reflect so-called ‘self-interestedness’, which means that they pursue their personal interest or benefit without considering others. This study examined how researchers in Zimbabwe perceive instances of self-interestedness among research collaborators, and how these perceptions differ according to their career stage and the nature of collaboration. An online survey of researchers in Zimbabwe was conducted to gather information about six instances of self-interestedness among research collaborators. The results show that Zimbabwean researchers involved exclusively in national collaboration reported greater degrees of collaborator self-interestedness than Zimbabwean researchers involved in international collaboration. However, early-career researchers and established researchers did not differ significantly regarding their experiences of collaborator self-interestedness. Measures aimed at capacitating research organisations and research teams in developing countries in the African context, to counter collaborator self-interestedness, are recommended.

## Introduction

Research collaboration is considered a key feature of any research system. Depending on the context and partners involved, such collaboration can take on different and often subtle forms, among which are transmission of know-how, provision of access to research equipment, mutual stimulation and trusted assessorship (Laudel, 2002). A more typical view of research collaboration – and the view reflected in this study – is that of a social process that involves interactions between two or more individuals or institutions, where the respective entities pool their human and scientific capital for the purpose of producing knowledge (Bozeman et al., 2013). Over the past decades, countless initiatives have been put in place to promote research collaboration between individuals and institutions across sectors and geographical boundaries. This is not surprising, given the general positive value attached to research collaboration (Katz & Martin, 1997). Nowadays, research collaboration is considered essential to bring about sustainable global change, as reflected in the United Nations Sustainable Development Goals (SDGs). For example, Goal 17 of the SDGs emphasises the need to revitalise global partnerships for sustainable development policies aimed at fostering global research partnerships and collaborations for sustainable development. An action plan set to achieve this goal is “to enhance North–South and South–South and triangular regional and international cooperation on and access to science, technology, and innovation …” (United Nations, 2022, p. 26).

Although generally associated with positive research outcomes, collaboration is a complex phenomenon because of the often unpredictable nature of human interactions between scientists (Subramanyam, 1983). As in all human interactions, occurrences of tensions, differences and challenges are inevitable (see Bozeman et al., 1999). Often these challenges stem from the self-interest of research collaborators, where they disregard others and pursue their own interests; in essence, going against so-called ‘disinterestedness’ as one of the norms of science (Merton, 1973, pp. 275–277). Merton proposed this norm (and three more –– universalism, communism, and organised scepticism) to describe what is known as the ethos of modern science. Disinterestedness expresses the idea that scientists should work only for the benefit of science and that scientific work should remain unspoiled by motivations of self-interest. However, due to a combination of factors in the broad research environment, and because scientists have different personalities and behaviour types, scientists may lack the ideal of disinterestedness in their collaborative practices.

The opposite of disinterestedness is self-interestedness, defined by the Oxford Dictionary as the pursuit of “one's personal interest or advantage, especially when pursued without regard for others” (Oxford Learner's Dictionary, n.d.). Researchers are expected to be professional in their work, trustworthy, and honest, have good relations with their colleagues, and be loyal to the organisation where they work (National Academy of Sciences, 2009). However, not all expectations always reflect reality. For instance, an environment in which scientific achievement is measured by the number of publications, combined with constant pressure to obtain funds for research – both of which are facilitated through collaboration – can create potential for the occurrence of unethical behaviour and a leaning towards self-interestedness (Edwards & Roy, 2017).

Against this background, the current study focuses on six instances of self-interestedness, among which undeserved or ‘gift’ authorship, publication without consent or offering others the chance to review the manuscript, and sharing research data without consent. A dual research question was addressed in the context of an African country: to what extent do researchers in Zimbabwe experience the six instances of collaborator self-interestedness as a problem in their own collaborations, and to what extent do their responses differ according to their research career stage and nature of research collaboration? Gender was also brought into the picture, not as a key focus, but by looking at the intersectionality between gender and both research career stage and nature of collaboration in the relationship with self-interestedness. An intersectional lens is often recommended to detect potential interconnecting injustices (Lynch et al., 2020).

Since the relationship between the perceived self-interestedness of collaborators and the research career stage of the observer is a key focus, as well as the relationship between self-interestedness and the nature of collaboration, brief discussions of these concepts and their possible relation to self-interestedness are presented next. Hypotheses are also stated. Some thoughts are also provided on the role of gender in the relationships, again with hypotheses. Before doing so, a brief note on Zimbabwe as the study's contextual focus is warranted.

## Zimbabwe as Contextual Focus

The case of Zimbabwe in southern Africa, as far as research collaboration is concerned, is an interesting one. Between the early 1900s and mid-2000s, the country went through a series of sociopolitical events that adversely affected its research system, especially since relations with most funding organisations had become strained. Prior to 2000, the country had relied on donor sources for much of its research funding. However, by 2003, most donors had suspended their operations due to political instability (Besada & Moyo, 2008), with many researchers seeking employment in neighbouring South Africa. Despite these challenges, some researchers in Zimbabwe continued to engage in international collaboration (see Ngwenya & Boshoff, 2020). Considering the country's generally strained relations with the international community, particularly western countries, a comparison of the experiences of researchers in Zimbabwe in different categories of collaboration is relevant. More information about the different facets of the research environment in Zimbabwe can be found elsewhere (e.g., Besada & Moyo, 2008: Boshoff & Ngwenya, 2022; Ndlovu et al., 2016; Ngwenya & Boshoff, 2018, 2020, 2022).

## Self-Interestedness in Research Collaboration in Relation to Research Career Stage

According to Arthur et al. (1989), a (research) career is the unfolding sequence of a person's (research) work experience over time. Recently, there has been a growing interest, both scientifically and politically, in the development of early-career researchers, as they “represent the future academic workforce” (Crome et al., 2019, p. 717). Descriptors such as ‘early-career researchers’, ‘emerging researchers’, ‘junior researcher’ or ‘next-generation researchers’, et cetera, all refer to the same phenomenon.

Scholars differ on the appropriate categorisation of an early-career researcher as different criteria can be used, such as qualification, age, length or stability of employment, research record, publications and previous grants (Bazeley, 2003). Bazeley's (2003, p. 274) own definition of an early-career researcher is:

Someone currently within their first five years of academic or other research-related employment allowing uninterrupted, stable research development following completion of their postgraduate research training.

This definition assumes that completion of postgraduate research training (typically a doctorate) is an essential foundation to build on, and that five years is sufficient time to start building a track record. However, it is not uncommon for the award of a doctorate to be followed by a period of unemployment in the case of some doctoral graduates. Someone may also have extensive work experience that is non-research related, and then only shift to research upon registration for a doctoral qualification. The definition by Friesenhahn and Beaudry (2014, p. 57) applies an age limit of 40 years by defining an early-career researcher as:

Someone of any discipline actively pursuing a research career, usually without being fully established yet. She/He will have received a PhD or an equivalent doctoral qualification up to 10 years ago and is usually between 30 and 40 years old.

However, an age limit of 40 years can be contested in the African environment. For example, in their analysis of young scientists in Africa, Beaudry et al. (2018) found that more than half (54%) of scholars who graduated with a doctorate in the preceding 10 years were over 40 years old. The average age at the time of doctoral graduation was 45 years. Beaudry and her colleagues also argue that scholars generally publish their first article at a younger age than at the age when they graduate with a doctorate. For example, some researchers with a master's degree would probably already have published a first article before registering for a doctoral qualification. This implies that there is no clear association between chronological age, graduation age and age at first publication in the African environment, and that self-classification may be an acceptable option for operationalisation of an early-career researcher.

It has been argued that emerging researchers are more likely to face collaborative challenges than established and senior researchers (Tsai et al., 2016; Youtie & Bozeman, 2016). Some of these challenges relate to research contribution issues and unequal authorship credit decisions. Bennett and Taylor (2003) note that junior researchers may feel pressured to give senior researchers undeserved credit to get published more easily, or to repay favours for funding and research opportunities. To make matters worse, senior researchers often have the power to distort authorship credits. Senior researchers can also abuse or bully their junior co-workers by distorting co-authorship credits or engaging in deceptive behaviour (Kwok, 2005). In a study by Tsai et al. (2016), some senior researchers did not consider students working with them on collaborative projects as collaborators; the senior researchers regarded themselves as the teachers rather than the collaborators of the students. In the same study, junior researchers, compared to their senior counterparts, emerged as facing greater pressures in terms of getting tenure, promotion, resources, and even academic prestige. Although the current study focused on whether junior researchers are more likely than established researchers to view self-interestedness as a challenge in their own collaboration, and not whether junior collaborators themselves act in self-interested ways, the latter is nonetheless a likely scenario. Given the lack of employment security of early-career researchers and the need to demonstrate their worth to keep their employment, they may at times feel a certain obligation to act in self-interested ways to establish their careers. In the current study, however, based on the mentioned literature, the assumption is that emerging researchers, compared to established researchers, are more likely to rate instances of collaborators reflecting self-interestedness as a serious problem in their own collaborations. It therefore was hypothesised:

Hypothesis 1: Early-career researchers will be significantly more likely than established researchers to rate instances of perceived self-interestedness as a major problem in their collaborations.

## Self-Interestedness in Research Collaboration in Relation to the Nature of Collaboration

Existing studies with a broad focus on collaborative experiences and challenges (e.g., Tsai et al., 2016; Youtie & Bozeman, 2016) rarely refer to collaborations involving African researchers, although studies focused on so-called ‘North-South’ research partnerships (e.g., Dodsworth, 2019; Ishengoma, 2016) do discuss African participation in the context of establishing equal partnerships. Insights into the types, patterns and nature of research collaboration in Africa, more broadly, are mainly gained through bibliometric studies (Adams et al., 2014; Mêgnigbêto, 2013; Mouton & Blanckenberg, 2018). In bibliometric studies, co-authorship of research publication is considered a proxy for research collaboration. Consequently, there is an established research interest in understanding co-authorship practices. Breet et al. (2018), for example, examined the co-authorship practices of South African researchers and found that 59% differed on who qualifies for co-authorship, while 48% had differences related to authorship sequence.

Some studies combine bibliometric co-authorship analysis with survey or interview analysis to understand the dynamics of research collaboration in the African context. While recognising that co-authorship does not necessarily equate to collaboration, such studies typically seek information on collaboration related issues from the authors of publications. For example, Boshoff (2009) used a survey to investigate the contributions that Cameroon co-authors had made to the collaborative research processes underlying internationally co-authored publications between researchers in Cameroon and three countries in the global ‘North’. To do this, the corresponding authors of publications in both Cameroon and the three international countries were surveyed. About 80% of international authors claimed that their Cameroonian co-authors had largely been involved in fieldwork, data collection and interpretation of results, implying a role distribution in which the Cameroonian authors are seen primarily as data providers. Similar role divisions were noted in a study by Owusu-Nimo and Boshoff (2017) on research collaboration in Ghana, which used the same methodology but surveyed only the Ghanaian corresponding authors.

What the various bibliometric studies on research collaboration in Africa also show is that African researchers collaborate more with researchers outside Africa than with researchers in Africa (Confraria et al., 2020; Guns & Wang, 2017). This preference is motivated by a need to gain access to research infrastructure and funding, and to build research capacities and scientific networks (Confraria et al., 2020). Moreover, the collaborations of African researchers are initiated by many factors, including shared research interests, previous collaboration, mutual acquaintances, student-supervisor interactions, existing interinstitutional partnership agreements, and funding agency requirements (Maluleka et al., 2016; Mouton et al., 2018; Owusu-Nimo & Boshoff, 2017).

In a mixed-methods study on research collaboration in Kenya, using a survey and interviews, Muriithi et al. (2018) brought to light factors that influence the organisation and conduct of academic research collaborations in Kenya. Three categories of challenges were identified: (i) problems of a sociocultural nature, comprising issues related to scientific competition, cultural differences, information security, conflict resolution, authorship inclusion and order, diverse disciplinary training of collaborators, and selection of a publication forum; (ii) problems of management and control, consisting of issues related to coordination of members’ activities, timely delivery of results, definition of roles, availability of time to commit to research, leadership and control, availability of skilled personnel, and administration of funding; and (iii) problems of availability of resources and the availability of and access to special equipment.

Many of the challenges faced by African researchers in international collaboration relate to structural power imbalances and asymmetric relationships between researchers in the so-called global ‘North’ and ‘South’. For example, Kombe et al. (2014) provide detailed narratives gathered from anecdotal evidence about unfair experiences facing researchers in the South when collaborating with those in the North. These include, among other things, insufficient recognition of the contribution of collaborators in the South and exclusion from final published manuscripts, even after playing crucial roles during the collaboration processes. An ethnographic study on “collaboration in transnational medical research from the viewpoint of African scientists working in partnerships with northern counterparts” (Okwaro & Geissler, 2015, p. 492) highlights some strategies used by African researchers in the field to ensure the sustainability of their research and collaboration despite persistent global research inequalities. Among the strategies are diversification of collaborators and research areas to optimise funding, and the maintenance of research sites to attract ongoing transnational activity.

The abovementioned impressions in the literature imply that the dynamics of international collaboration present certain challenges for African researchers. These impressions informed the current study's expectations of how serious a problem collaborator self-interestedness would be for Zimbabwean researchers in different collaboration categories. Given the power imbalances associated with international collaboration, it therefore was expected that Zimbabwean researchers participating in international collaboration (as opposed to those participating in national collaboration) would perceive self-interestedness by collaborators as a more serious problem. It therefore was hypothesised:

Hypothesis 2: Researchers in international collaboration will be significantly more likely than researchers in national collaborations to rate instances of perceived self-interestedness as a major problem in their collaborations.

## Gender as an Intersectional Variable

The current study focused on the self-interestedness of research collaborators as perceived by researchers in Zimbabwe, and whether such perceptions differed according to the nature of collaboration (i.e., national versus international collaboration) and the research career stage of Zimbabwean researchers. Since gender is used as an intersectional variable in these relations, it would be of interest to reflect on gender differences in research more broadly.

Several studies have reported gender asymmetries in various academic-related activities (Araújo et al., 2017; Baobeid et al., 2022; Beaudry & Larivière, 2016; Kwiek & Roszka, 2021; Larivière et al., 2013). Most of these have been carried out outside Africa and the focus has been on asymmetries in relation to issues such as hiring, grant funding, research production and collaboration strategies. It is reported that women scientists occupy more junior positions, have lower salaries, are more often in non-tenure-track and teaching-only positions, and receive less grant money compared to their male counterparts. For example, Larivière et al. (2011) showed that, in Quebec, Canada, women raised less research funds and their funding was less diversified compared to that of men. In terms of research productivity, men also publish more papers on average than women (Larivière et al., 2013).

A study of the collaboration patterns of African researchers showed that almost similar percentages of women and men participate in collaboration, whether intra-institutional, national or international (Mouton et al., 2018). However, women are said to be less likely to participate in collaborations leading to publication, and are less likely to be listed in prominent author positions. For example, in a study analysing the extent to which female researchers in health research in sub-Saharan Africa hold prestigious authorship positions (first or last author, or sole author), Baobeid et al. (2022) found higher shares of male authors compared to female authors in all the prestigious positions. Articles with women in dominant author positions also received fewer citations than those with men in the same positions (Larivière et al., 2013).

Men and women also differ significantly in their strategies for choosing associates. Bozeman and Gaughan (2011) found that, compared to their male counterparts, female faculty researchers were less oriented towards collaboration based on instrumentality (that is, collaboration concerned with immediate work factors, including the allocation of credit) or collaboration based on past collaborative experiences. Under various circumstances, female researchers also seem to work more closely with other women (Bozeman & Corley, 2004). Moreover, in a study by Gaughan and Bozeman (2016), both male and female faculty had experienced and observed past dominance and exploitation by senior faculty of both sexes. The same study also found gender and seniority to be intertwined in relation to constructs such as personality, style and interpersonal collaboration dynamics. However, since seniority in research teams generally tends to be vested in the male members of such teams, both female and male early-career researchers may be equally likely to view self-interestedness as a major problem in their collaboration (assuming the self-interested individuals are the senior staff members). The following was therefore hypothesised regarding the relationship between career stage and perceived self-interestedness of research collaborators, using gender as an intersectional variable:

Hypothesis 3a: Female and male early-career researchers will not differ significantly in their rating of instances of perceived self-interestedness as a major problem in their collaborations.

Hypothesis 3b: Female and male early-career researchers will be significantly more likely than female and male established researchers to rate instances of perceived self-interestedness as a major problem.

Furthermore, the literature shows that female researchers generally tend to appear in a position of disadvantage compared to their male counterparts. It is also hypothesised elsewhere in this study that researchers in international collaborations would be more likely than researchers in national collaborations to consider self-interestedness as a major problem. Logically, then, the following was hypothesised about the relationship between the nature of collaboration and perceived self-interestedness of research collaborators, with gender as an intersectional variable:

Hypothesis 4a: Female researchers in international collaborations will be significantly more likely than male researchers in international collaborations to rate instances of perceived self-interestedness as a major problem in their collaborations.

Hypothesis 4b: Female and male researchers in international collaborations will be significantly more likely than female and male researchers in national collaborations to rate instances of perceived self-interestedness as a major problem.

## Method

The method employed was an online survey of researchers in Zimbabwe, focused on instances of self-interestedness among research collaborators.

### Survey Questionnaire

The questions on self-interestedness were taken from a questionnaire developed for the doctoral study of the first author, and the questionnaire was compiled by considering various sources (e.g., Bozeman et al., 2016; Bozeman & Corley, 2004; Bozeman & Gaughan, 2011). The questionnaire consists of four sections, but for this study on self-interestedness only parts of two sections were used (sections one and four). All four sections are mentioned here briefly for context. The first section seeks to solicit information about the collaboration activities of researchers in Zimbabwe, for example information on how often researchers engage in collaboration, with whom they collaborate, reasons for collaborating, how collaborations are initiated, and challenges encountered during collaboration. The second section focuses on article authorship by soliciting information on authorship disputes in the past, how the disputes came about and how they were resolved. The third section focuses on data ownership and data sharing, gathering information about past disputes related to data ownership, if any, and how they were resolved. The last section gathers information on the demographic details of the survey respondents. The complete questionnaire appears in the doctoral study, which is available in an online repository (Ngwenya, 2021). The doctoral study itself was primarily a bibliometric study and the survey was used to supplement the bibliometric results.

The first section solicited information about the collaboration activities of researchers in Zimbabwe. For example, on a five-point rating scale (‘always’ [1], ‘often’ [2], ‘sometimes’ [3], ‘seldom’ [4] and ‘never’ [5]), the respondents had to indicate how regularly they engaged in research collaboration. They were also asked to specify, among others, the frequency of collaboration with researchers in different geographical locations, and to rate various statements about challenges encountered in research collaboration in terms of severity. This study focused on six of those statements, all of them considered examples of the self-interestedness of collaborators. On a four-point rating scale (‘not applicable/no problem at all’ [0], ‘a minor problem’ [1], ‘a moderate problem’ [2], and ‘a major problem’ [3]), the respondents rated how serious a problem each of the following statements was in their own research collaborations:

- Collaborators using or distributing the research data without informing others
- Collaborators publishing the group's work without informing others
- Collaborators not sharing relevant information
- Collaborators insisting on co-authorship without having made any contributions
- Collaborators not delivering work when and/or as agreed
- Collaborators acting only in their own interests without considering others

The six statements above are indicative of four interpretations of self-interestedness. The first has to do with co-workers *disregarding the need for consent in a collaboration*. Statements one and two capture this interpretation, specifically in relation to the sharing of research data and publishing. However, various reflections on these two statements are possible. The act of self-interestedness in question may not be so much a breach of consent by commission (collaborators feeling entitled to do what serves their own best interests), but also a breach of consent by omission (collaborators accidentally forgetting that co-worker consent is required when sharing group outputs). In any case, whatever the true intent of the individual concerned might have been, from an outsider perspective both possibilities reflect self-interestedness. The second aspect of self-interestedness, captured by the third statement, is the *withholding of relevant information from co-workers*. As before, the motivations for this act of self-interestedness remain open to interpretation and can perhaps best be explored through an in-depth case study, which was beyond the scope of this exploratory survey.

*Undeserved or ‘gift’ authorship* is a third aspect, addressed by statement four. Again, the meaning of ‘undeserved’ is open to different interpretations. For example, some contributions could have been made ‘behind the scenes’, such as where the self-interested person, unknown to the respondent, obtained funding for the study or wrote a protocol. However, the fact that such an arrangement took place ‘behind the scenes’, or was known only to the individual accused of self-interestedness or to a select few in the team, means that the collaboration may not be on a healthy footing. Such collaboration lacks transparency in the first place, and the insistence of the alleged self-interested person to be included as a co-author is what makes this statement an example of self-interestedness. The fourth issue, about *agreed upon timelines being ignored*, is at the intersect between disrespect for prior arrangements and serving one's own interests. Although there may be good reasons on the part of the self-interested person for not delivering work as agreed (poor time management, other pressing priorities and so on), it is not the reasons for a perceived act of self-interestedness that are relevant in this study, but the perceived severity of the problem in research collaboration. Finally, a generic and overarching statement of self-interestedness was also included, as reflected in the wording of the sixth and last statement included in the survey.

The other section of the original questionnaire used in this study of self-interestedness was the demographic one. It collected details of the survey respondents, specifically their gender, age, sector affiliation and field of research. Since it is generally argued that, when researchers study abroad, they tend to maintain connections with their research teams even after returning to their home countries, the survey respondents were also asked to indicate from where they obtained their highest qualification. Lastly, the respondents were asked to classify themselves in one of four mutually exclusive research career categories, taken from the European Framework for research careers (European Commission, 2011):

- First stage researcher (not yet a PhD or still busy with a PhD)
- Recognised researcher (PhD holder or equivalent who is not yet fully independent)
- Established researcher (researcher who has developed a level of independence)
- Leading researcher (researcher leading his/her own research area or field).

In the present study, a binary classification of career stage was used, where the first two categories above were combined to form a new category of ‘early-career researcher’ (ECR), and the last two to create a category of ‘middle- to late-career researcher’ (MLCR).

### Survey Administration

The e-mail addresses of Zimbabwean researchers (for use in an online survey conducted in November 2018) were obtained from available article information. Specifically, the contact details of Zimbabwean authors with articles published between 2012 and 2016 were downloaded from three bibliographic databases: Web of Science (WoS), Scopus, and the National Research Database of Zimbabwe (NRDZ). The NRDZ is “an online integrated and comprehensive ‘one stop shop’ covering all public domain research” in the country (RCZ, 2020). The e-mail addresses collected from articles were supplemented with e-mail addresses from the websites of universities and research organisations in Zimbabwe. All e-mail addresses of academic and research staff with at least a master's degree were captured. In total, 6 160 non-unique e-mail addresses were collected – 1 212 were from websites, 2 412 from Scopus, 2 169 from WoS and 367 from the NRDZ. After removing all duplicates, 3 046 unique e-mail addresses remained.

A link to access the online questionnaire, along with a cover letter, was sent to the 3 046 Zimbabwean e-mail addresses. Of these, 654 (21.4%) bounced back. This means that 2 392 e-mails reached their target. This figure was considered a good estimate of the total research workforce in Zimbabwe, given that 2 739 researchers were recorded in the country in 2012, based on the results of the latest survey on research and development in Zimbabwe (United Nations Educational, Scientific and Cultural Organization [UNESCO], 2014). A total of 316 researchers responded to the questionnaire, which means a response rate of 13% (out of 2 392).

### Survey Respondents

The demographic details of the survey respondents are presented in Table 1. The largest percentage of respondents were male (75%), between 35 and 44 years old (43%), with a master's degree as their highest academic qualification (53%), an early-career researcher (ECR, 67%), from the university sector (82%), and in the field of social sciences and humanities (45%).

**Table 1.** Demographic Details of Survey Respondents.

Demographics	Count	%	Demographics	Count	%
**Gender**			**Sector**		
Female	59	25%	University	190	82%
Male	178	75%	Government organisation	14	6%
			Public research organisation	10	4%
**Age**			Non-governmental organisation	12	5%
25–34 years	37	17%	Industry/business	7	3%
35–44 years	91	43%			
45–55 years	59	28%	**Research field**		
55+ years	27	13%	Agricultural sciences	24	10%
			Health sciences	48	21%
**Highest academic qualification**			Natural sciences	55	24%
Master's degree	121	53%	Social sciences and humanities	105	45%
Doctoral degree	109	47%			
**Research career stage**					
ECR	157	67%			
MLCR	79	33%			

*Note*. Some distributions in Table 1 are similar to those reported by other studies. For example, a survey on research and development in Zimbabwe, conducted by United Nations Educational, Scientific and Cultural Organization (UNESCO) in 2012, reported that 25% of researchers in the country were female (UNESCO, 2014), the same as in Table 1. The UNESCO study further reported that 92% of Zimbabwean researchers were operating in the university sector (compared to 82% in the current study). However, as the UNESCO study surveyed only university- and government-based researchers, the figures in Table 1 were adjusted by excluding three of the five sector categories to generate a new sector-based total. Subsequently, in the current study, 93% of researchers (of those in the university and government sectors only) were found to be in the university sector (about the same as in the UNESCO study). However, whereas the UNESCO study, in 2012, reported that 81% of researchers had a master's degree, in the current study it was 53%.

‘Public research organisation’ refers to a public entity that is involved in research activities, but which is not a university or a government organisation.

### Data Analysis

Data were analysed using a statistical analysis software programme, namely SPSS Version 28. The analyses were largely descriptive, but statistically significant testing was also used. Since the variables in the analysis were all categorical in nature, a Pearson chi-square test of independence was used to determine whether any two variables were significantly related. Where a chi-square test produced a significant result, i.e., the null hypothesis of independence was rejected, a set of post-hoc tests were performed. The purpose of the follow-up tests was to determine which proportions in a cross-tabulation were significantly different from each other, and thus responsible for the overall significance as found by chi-square. The appropriate post-hoc test was a pairwise Z-test, with p-values adjusted according to the Bonferroni method. The reason for the adjustment was that, when multiple pairwise tests are applied in a single analysis, the probability of false positives increases. Bonferroni corrects for the number of pairwise tests by dividing the critical p-value by the number of comparisons made. In cases where the assumptions for performing a chi-square test were violated due to too many small cell values, a Fisher-Freeman-Halton exact test was used as an alternative.

## Results

The results are presented in four sections. The first shows the ratings of severity of self-interestedness of research collaborators, as perceived by Zimbabwean researchers. The next section provides a general overview of research collaboration by Zimbabwean researchers, as well as profiles of Zimbabwean researchers in each of two categories of research collaboration (national and international). The third section provides profiles of Zimbabwean researchers in each of two research career stages (ECR and MLCR). The last section investigates the relationships between perceived self-interestedness among collaborators, and the nature of research collaboration and the research career stage of the Zimbabwean researchers, respectively.

### Self-Interestedness of Collaborators

**Figure 1.**
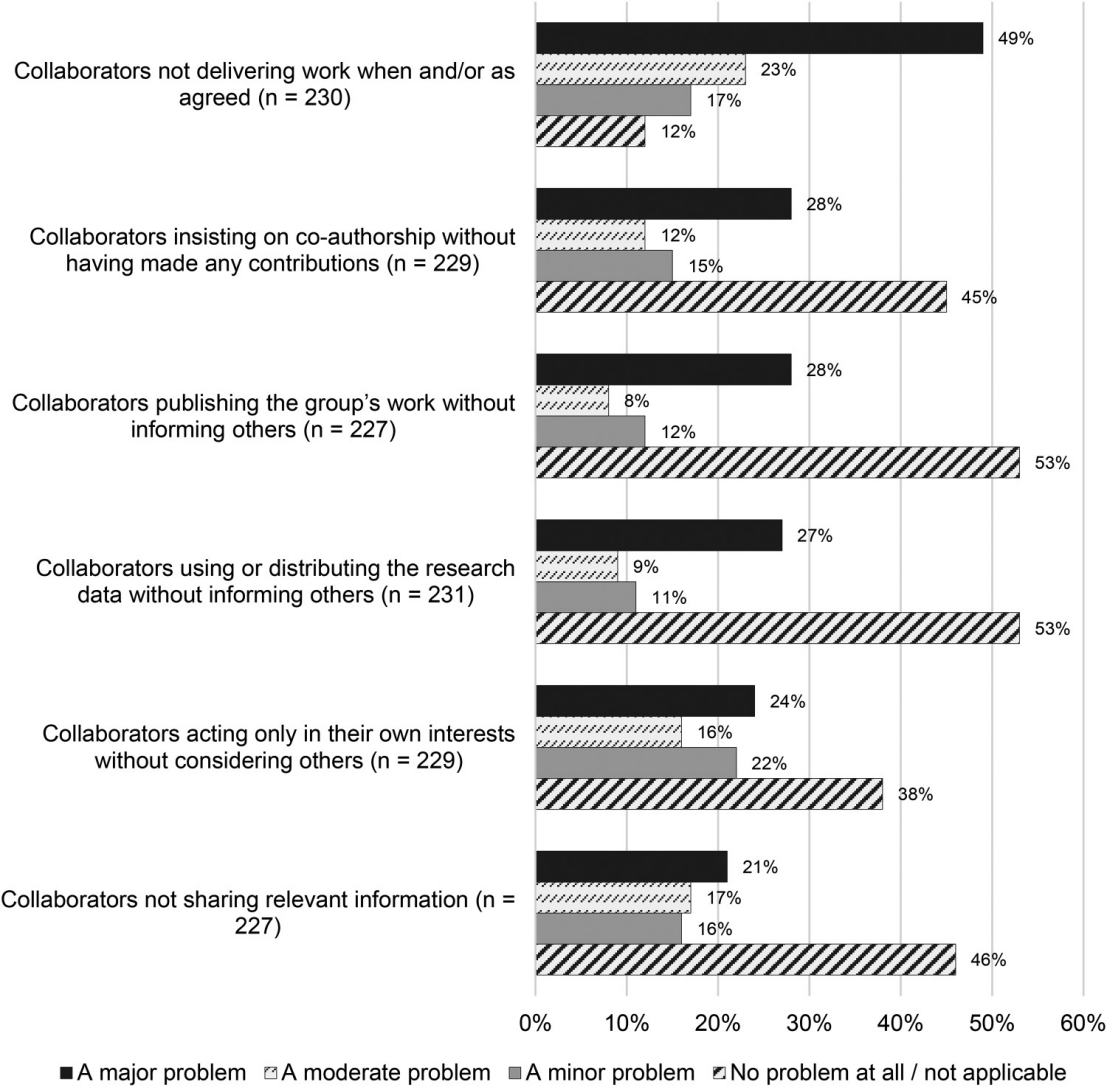
Percentage distribution of responses to six instances of self-interestedness of collaborators.

 Figure 1 shows findings for the six examples of self-interestedness of research collaborators. The rating or level of severity was based on the Zimbabwean researchers’ own collaborative experiences as a point of reference. The example most often rated as a major problem was one of the collaborators not delivering work when and/or as agreed. Of a total of 230 respondents, almost half (49%) of the respondents considered the severity of this problem as ‘major’, with 23% perceiving it as ‘moderate’ and 17% as ‘minor’. Moreover, 28% of the respondents regarded collaborators who insisted on co-authorship without having made any contributions as a major problem in their own research collaborations. The same rating of severity (a ‘major problem’) also applied to two other instances of self-interestedness, namely collaborators who published the group's work without informing others (28%) and collaborators who used or distributed the research data without informing others (27%). However, it cannot be ignored that large percentages of respondents (38% to 53%) regarded five of the six instances of self-interestedness as either no problem in their collaboration, or not applying to them at all.

### Research Collaboration

The survey respondents were asked to indicate how regularly they engaged in research collaboration, where collaboration can refer to either participation in joint research or the production of joint publications. A total of 259 researchers responded to the question. Of this total, a small fraction (23 or 9%) said that they seldom or never engaged in research collaboration; the rest (236 or 91%) indicated that they do engage in research collaboration (i.e., ‘always’, ‘often’ and ‘sometimes’ responses). The 236 were subsequently asked to specify the geographic locations of the individuals with whom they collaborate. Table 2 shows, for those who responded, the percentage distribution of their frequency of collaboration with individuals in eight geographic locations.

**Table 2.** Percentage Distribution of Frequency of Collaboration, Either in Joint Research or Through Joint Publications, with Individuals in Eight Geographic Locations.

Geographic locations	Count	Frequency of collaboration			
		Always	Often	Sometimes	Never
Own organisation in Zimbabwe	227	30%	40%	22%	8%
Other organisations in Zimbabwe	228	7%	25%	41%	27%
South Africa	224	4%	18%	33%	45%
Rest of Africa (South Africa excluded)	226	2%	8%	27%	62%
Europe	225	3%	9%	24%	64%
United States of America	218	2%	7%	14%	77%
Asia	217	2%	2%	6%	90%
Elsewhere in the world	220	2%	1%	16%	81%

Most respondents (92% of 227) collaborated with researchers from within their organisations in Zimbabwe (the categories ‘always’, ‘often’ and ‘sometimes’ combined indicate collaboration). Notable percentages of respondents also collaborated with researchers in other organisations in Zimbabwe (63%), as well as with researchers in South Africa (55%). Collaboration with researchers from outside Africa is relatively less common, with European collaboration being the most prominent (36%).

The eight geographical locations in Table 2 each correspond to a separate variable in the underlying dataset. The eight variables were combined into a single variable in the dataset, consisting of three mutually exclusive categories: ‘national collaboration only’ (29%), ‘international collaboration only’ (6%), and ‘both national and international collaboration’ (65%). Given the small number of respondents in the ‘international collaboration only’ category, this new variable was in turn modified to reflect two categories: ‘national collaboration only’ (29%) and ‘international collaboration with or without national collaboration’ (71%). This two-category variable of collaboration was the one considered in relation to the six instances of self-interestedness. However, before this was done, the two categories of collaboration were first profiled in terms of the characteristics of the researchers in each category (Table 3). The reason for the profiling was to understand what the researcher characteristics look like in each category of collaboration (in terms of age, gender, country of highest degree, etc.), and whether the categories differ significantly from each other in terms of those characteristics. Chi-square tests, along with follow-up pairwise Z-tests applying the Bonferroni correction, were used for testing statistical significance. Information about the composition of each comparison group, and whether they differed significantly from each other, was considered informative for interpreting a potentially significant relationship between the collaboration variable and the instances of self-interestedness.

**Table 3.** Profiling two Categories of Research Collaboration in Terms of Researcher Characteristics.

Researcher characteristics	National collaboration [A]	International collaboration [B]	Significance testing	
			Overall significant association	Significant pairwise group differences
**Age**	*(n = 55)*	*(n = 140)*	* *	* *
25–34 years	16%	17%	χ^2^ = 1.306, *p* = .728	Not applicable
35–44 years	38%	46%		
45–54 years	31%	26%		
55 + years	15%	11%		
**Gender**	*(n = 61)*	*(n = 154)*	* *	* *
Female	16%	29%	χ^2^ = 3.445, *p* = .063	Not applicable
Male	84%	71%		
**Country of highest degree**	*(n = 59)*	*(n = 147)*	* *	* *
Zimbabwe	75%	35%	χ^2^ = 26.046, *p* < .05	A > B
South Africa	12%	33%		B > A
Rest of world	14%	32%		B > A
**Field**	*(n = 57)*	*(n = 153)*	* *	* *
Agricultural sciences	5%	14%	χ^2^ = 19.578, *p* < .05	None
Health sciences	11%	26%		B > A
Natural sciences	18%	27%		None
Social sciences and humanities	67%	33%		A > B
**Career stage**	*(n = 60)*	*(n = 154)*	* *	* *
ECR	78%	60%	χ^2^ = 6.145, *p* < .05	A > B
MLCR	22%	40%		B > A
**Team size (self included)**	*(n = 68)*	*(n = 166)*	* *	* *
2 individuals	15%	9%	χ^2^ = 22.369, *p* < .05	None
3 individuals	40%	20%		A > B
4 individuals	31%	26%		None
5 individuals	7%	14%		None
≥ 6 individuals	7%	31%		B > A

*Note*. ‘National collaboration’ means ‘national collaboration only’, and ‘international collaboration’ means ‘international collaboration with or without national collaboration’.

Table 3 shows, for each category of collaboration, the percentage distribution of survey respondents in terms of their demographics. As can be seen, the respondents in the two categories of collaboration do not differ significantly in terms of age and gender. However, they differ significantly in terms of career stage and the country where the highest degree was obtained. Three-quarters (75%) of respondents involved in national collaboration obtained their highest qualification in Zimbabwe, compared to 35% of respondents involved in international collaboration. In terms of career stage, although both collaboration groups include large shares of ECRs (78% and 60% respectively), the share of ECRs is significantly higher for those involved in national collaboration only (78%).

The two categories of research collaboration were also profiled in terms of their constitutive parts, specifically the two elements of national collaboration (collaboration with own or other organisations in Zimbabwe) that informed the category development. The results are shown in Table 4. Accordingly, both the national collaboration group and the international collaboration group reported very high levels of collaboration with their own organisation (98% and 89%). These two percentages differ significantly, with the national group significantly more prone to collaboration within their own organisation in Zimbabwe. In turn, collaboration with other organisations in Zimbabwe was found to be significantly higher among the international group (79%) compared with the national group (59%), although both groups show high levels of such collaboration.

**Table 4.** Profiling two Categories of Research Collaboration in Terms of National Collaboration Patterns.

National collaboration patterns	National collaboration [A]	International collaboration [B]	Significance testing	
			Overall significant association	Significant pairwise group differences
**Own organisation in Zimbabwe**	*(n = 67)*	*(n = 160)*	* *	* *
Yes	98%	89%	χ^2^ = 5.862, *p* < .05	A > B
No	2%	11%		B > A
**Other organisations in Zimbabwe**	*(n = 69)*	*(n = 159)*	* *	* *
Yes	59%	79%	χ^2^ = 8.956, *p* < .05	B > A
No	41%	21%		A > B

*Note*. ‘National collaboration’ means ‘national collaboration only’, and ‘international collaboration’ means ‘international collaboration with or without national collaboration’.

### Research Career Stage

As indicated in the methods section, a binary classification of career stage was used, namely ECR (67%) and MLCR (33%). Table 5 profiles the two research career stages of the survey respondents in terms of researcher characteristics. As expected, the researchers in the two categories differed significantly in terms of age. The ECR category includes significantly more researchers in the two youngest age groups (23% vs. 6%, and 49% vs. 30%), while the MLCR group includes more researchers in the two oldest age groups (37% vs. 23%, and 28% versus 5%). Although there is a clear relationship between age and the ECR/MLCR classification, it is far from a perfect relationship. This is evident in that 49% of ECRs were between 35 and 44 years old, and 28% were 45 years and older. A lesson that can be drawn from this is that the use of chronological age as a criterion for classifying an early-career researcher should be done with caution, especially in the context of researchers in Africa.

**Table 5.** Profiling two Research Career Stages in Terms of Researcher Characteristics.

Researcher characteristics	ECR [A]	MLCR [B]	Significance testing	
			Overall significant association	Significant pairwise group differences
**Age**	*(n = 143)*	*(n = 71)*	* *	* *
25–34 years	23%	6%	χ^2^ = 36.062, *p* < .05	A > B
35–44 years	49%	30%		A > B
45–55 years	23%	37%		B > A
55 + years	5%	28%		B > A
**Gender**	*(n = 157)*	*(n = 79)*	* *	* *
Female	26%	24%	χ^2^ = 0.057, *p* = .811	Not applicable
Male	74%	76%		
**Country of highest degree**	*(n = 153)*	*(n = 75)*	* *	* *
Zimbabwe	59%	24%	χ^2^ = 29.795, *p* < .05	A > B
South Africa	26%	32%		None
Rest of world	18%	44%		B > A
**Field**	*(n = 153)*	*(n = 79)*	* *	* *
Agricultural sciences	10%	11%	χ^2^ = 3.048, *p* = .384	Not applicable
Health sciences	20%	26%		
Natural sciences	21%	29%		
Social sciences and humanities	49%	38%		
**Team size (self included)**	*(n = 140)*	*(n = 73)*	* *	* *
2 individuals	12%	7%	χ^2^ = 2.093, *p* = .719	Not applicable
3 individuals	24%	30%		
4 individuals	27%	27%		
5 individuals	13%	11%		
≥ 6 individuals	24%	25%		

Table 5 also shows that the survey respondents differed significantly in terms of where their highest degree was obtained. Significantly more ECRs (59%) than MLCRs (24%) obtained their highest degree in Zimbabwe. In turn, more MLCRs (44%) than ECRs (18%) reported that their highest degree was obtained in a country that is neither Zimbabwe nor the neighbouring South Africa. These results also partly explain the collaboration patterns in Table 6, where MLCRs collaborate significantly more with individuals outside Zimbabwe compared to ECRs. The finding provides support for the argument that researchers who have studied abroad tend to maintain ties with their international colleagues even after returning to their home countries.

**Table 6.** Profiling two Research Career Stages in Terms of Collaboration Patterns.

Collaboration patterns	ECR [A]	MLCR [B]	Significance testing	
			Overall significant association	Significant pairwise group differences
**Own organisation in Zimbabwe**	*(n = 137)*	*(n = 71)*	* *	* *
Yes	92%	92%	χ^2^ = 0.011, *p* = .916	Not applicable
No	8%	8%		
**Other organisations in Zimbabwe**	*(n = 137)*	*(n = 73)*		
Yes	69%	81%	χ^2^ = 3.590, *p* = .058	Not applicable
No	31%	19%		
**South Africa**	*(n = 133)*	*(n = 72)*		
Yes	50%	71%	χ^2^ = 8.577, *p* < .05	B > A
No	50%	29%		A > B
**Rest of world**	*(n = 140)*	*(n = 74)*		
Yes	49%	70%	χ^2^ = 9.254, *p* < .05	B > A
No	51%	30%		A > B
**Overall collaboration pattern**	*(n = 140)*	*(n = 74)*		
National collaboration	34%	18%	χ^2^ = 6.145, *p* < .05	A > B
International collaboration	66%	82%		B > A

*Note*. ‘National collaboration’ means ‘national collaboration only’, and ‘international collaboration’ means ‘international collaboration with or without national collaboration’.

Having profiled the research collaboration patterns and research career stages of the survey respondents, the focus now shifts to investigations of the relationship between collaborator self-interestedness, and research collaboration and research career stage, respectively.

### Relationship Between Perceived Self-Interestedness of Collaborators and the Nature of Research Collaboration of Zimbabwean Researchers

Table 7 shows how the respondents in the two categories of collaboration rated each item of self-interestedness in terms of severity. Statistically significant differences were found for three of the six items, two of which speak to disregarding the need for consent in a collaboration (research data used or shared without informing others, and the group's work published without informing others), and one to unearned or ‘gift’ authorship. For all three items, researchers involved in national collaboration considered the self-interestedness of collaborators to be a more serious problem compared to those involved in international collaboration. For example, 42% of respondents involved in national collaboration rated ‘collaborators insisting on co-authorship without having made contributions’ as a major problem, while only 23% involved in international collaboration rated the same issue as a major problem. Similarly, a higher share (43%) of respondents involved in national collaboration compared to a relatively smaller share (21%) of those involved in international collaboration rated ‘collaborators using or distributing the research data without informing others’ as a major problem.

**Table 7.** Severity Ratings for six Instances of Self-Interestedness in National Versus International Research Collaboration<>.

Instances of self-interestedness	National vs international collaboration	Rating of severity				Significance testing
		Major problem	Moderate problem	Minor problem	No problem	
Collaborators not delivering work when and/or as agreed	National	55%	24%	9%	12%	χ^2^ = 4.135 *p* = .247
	International	46%	23%	20%	12%	
Collaborators insisting on co-authorship without having made contributions	National	42%	16%	10%	31%	χ^2^ = 12.871 *p* < .05
	International	23%	10%	17%	50%	
Collaborators publishing the group's work without informing others	National	41%	9%	8%	42%	χ^2^ = 9.410 *p* < .05
	International	22%	9%	13%	58%	
Collaborators using or distributing the research data without informing others	National	43%	8%	8%	42%	χ^2^ = 12.301 *p* < .05
	International	21%	10%	12%	57%	
Collaborators acting only in their own interests without considering others	National	28%	18%	19%	34%	χ^2^ = 1.514 *p* = .679
	International	22%	15%	23%	40%	
Collaborators not sharing relevant information	National	28%	16%	15%	40%	χ^2^ = 3.126 *p* = .363
	International	18%	17%	16%	49%	

*Note*. Highlighted cells (dark frames) mean that the two values compared are significantly different (*p* < .05) based on a pairwise Z-test, with the critical p-value adjusted according to the Bonferroni method.

**Figure 2.**
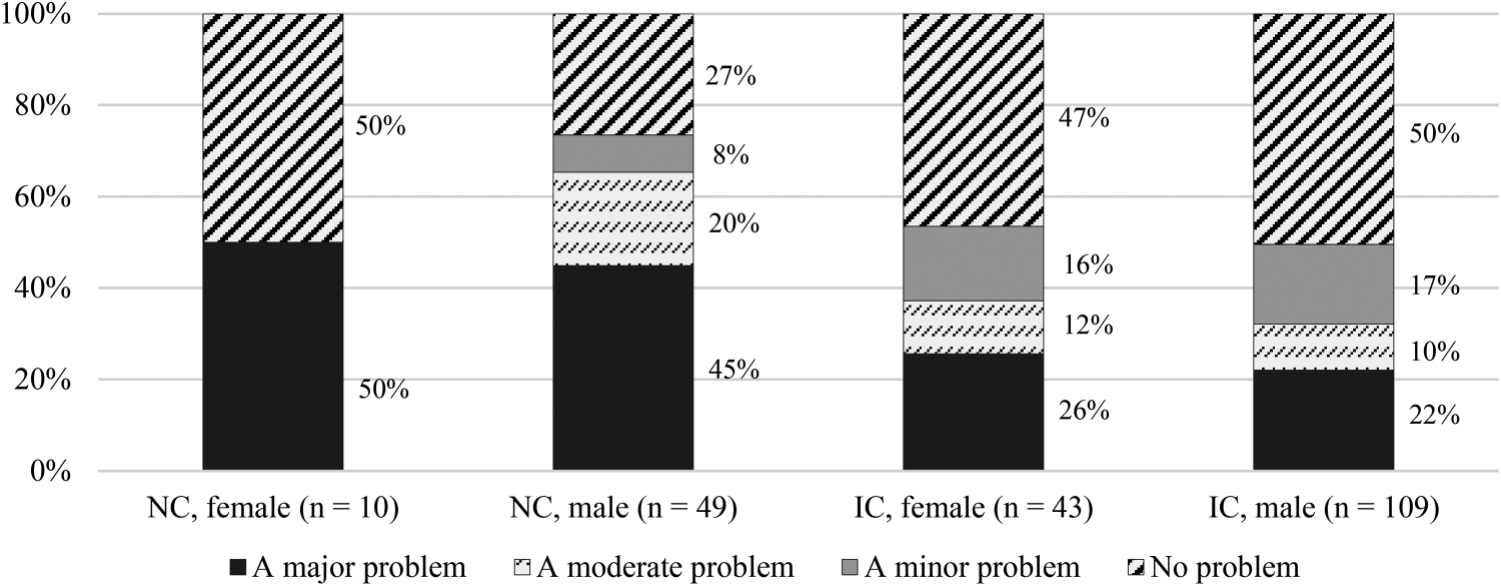
Severity ratings for the statement, ‘Collaborators insisting on co-authorship without having made contributions’, by nature of collaboration and gender. *Note*. The Fisher-Freeman-Halton exact test revealed a statistically significant association between the intersectional variable (nature of collaboration by gender) and the rating of severity for the relevant item (test value = 18.826, *p* < .05). A pairwise Z-test with the critical p-value adjusted according to the Bonferroni method was used as a follow-up test. NC = national collaboration; IC = international collaboration.

**Figure 3.**
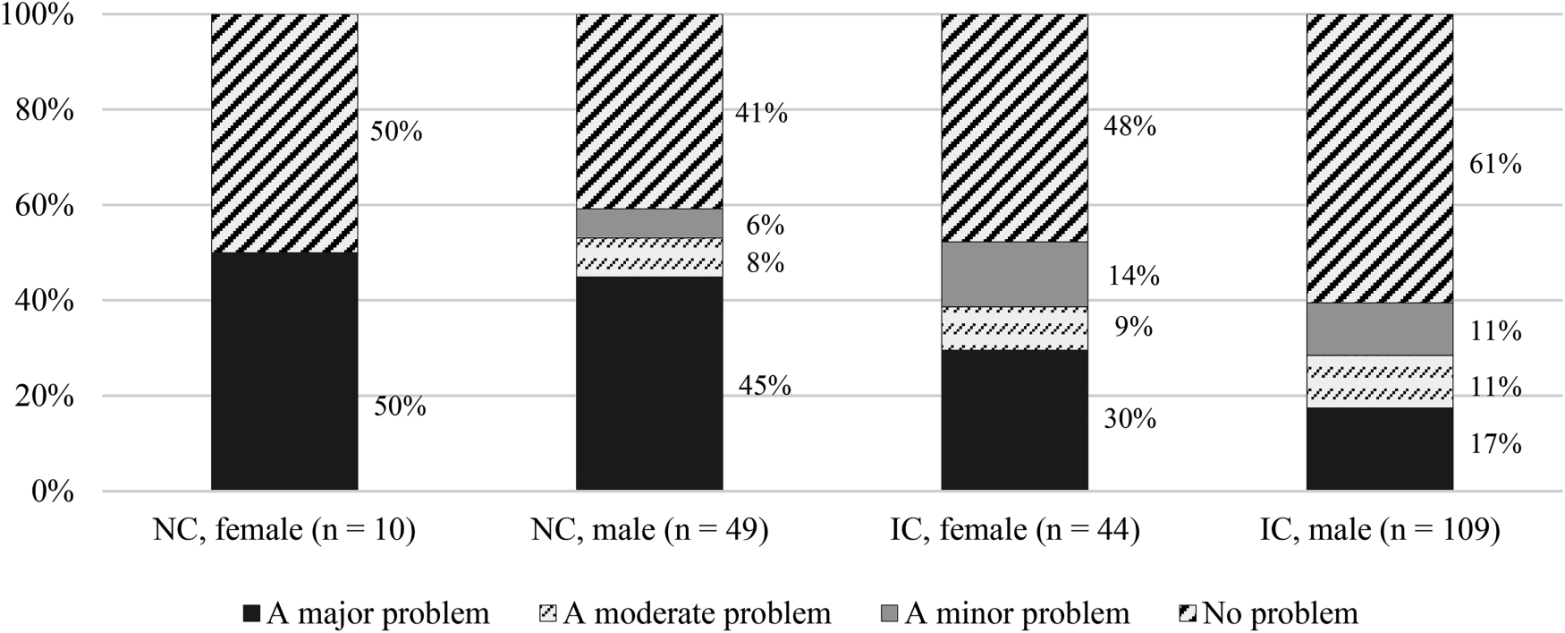
Severity ratings for the statement, ‘Collaborators using or distributing the research data without informing others’, by nature of collaboration and gender. *Note*. The Fisher-Freeman-Halton exact test revealed a statistically significant association between the intersectional variable (nature of collaboration by gender) and the rating of severity for the relevant item (test value = 16.232, *p* < .05). A pairwise Z-test with the critical p-value adjusted according to the Bonferroni method was used as a follow-up test. NC = national collaboration; IC = international collaboration.

The analyses in Table 7 were extended by including gender as an intersectional variable to determine whether any of the items of self-interestedness were significantly associated with a new collaboration-by-gender variable. Only two of the six cross-tabulations yielded an overall statistically significant association, and these cross-tabulations are displayed in Figures 2 and 3 respectively.

Figure 2 illustrates the statistically significant association between collaboration by gender and the item, ‘collaborators insisting on co-authorship without having made contributions’. Only two statistically significant group differences were responsible for the overall significance, as revealed by a pairwise Z-test with Bonferroni corrections. Male researchers involved in national collaborations (45%) were significantly more likely than male researchers in international collaborations (22%) to rate unearned or ‘gift’ authorship as a major problem. The reverse also applied: male researchers in international collaborations (50%) were significantly more likely than male researchers in national collaborations (27%) to view the issue in question as not being a problem. In Figure 3, the significant relationship is between collaboration by gender and the item, ‘collaborators using or distributing the research data without informing others’. Only one significant group difference emerged from a post-hoc pairwise Z-test: compared to male researchers in international collaborations (17%), male researchers in national collaborations (45%) were significantly more likely to view the use of research data without consent as a major problem in their own collaborations.

### Relationship Between Perceived Self-Interestedness of Collaborators and the Career Stage of Zimbabwean Researchers

Table 8 shows how the respondents in the two research career stages rated each of the six items of self-interestedness in terms of severity. None of the cross-tabulations were found to be statistically significant. This means that ECRs and MLCRs did not have significantly different perceptions of the severity of collaborator self-interestedness in their respective research collaborations.

**Table 8.** Severity Ratings for six Instances of Self-Interestedness by Career Stage.

Instances of self-interestedness	Career stage	Rating of severity				Significance testing
		Major problem	Moderate problem	Minor problem	No problem	
Collaborators not delivering work when and/or as agreed	ECR	50%	21%	18%	11%	χ^2^ = 1.644 *p* = .649
	MLCR	47%	25%	14%	15%	
Collaborators insisting on co-authorship without having made contributions	ECR	33%	10%	15%	41%	χ^2^ = 4.627 *p* = .201
	MLCR	22%	16%	12%	49%	
Collaborators publishing the group's work without informing others	ECR	29%	5%	13%	53%	χ^2^ = 5.167 *p* = .160
	MLCR	27%	13%	7%	54%	
Collaborators using or distributing the research data without informing others	ECR	30%	7%	9%	55%	χ^2^ = 4.535 *p* = .209
	MLCR	25%	15%	11%	49%	
Collaborators acting only in their own interests without considering others	ECR	26%	15%	19%	40%	χ^2^ = 2.848 *p* = .416
	MLCR	22%	19%	26%	32%	
Collaborators not sharing relevant information	ECR	22%	16%	19%	45%	χ^2^ = 0.519 *p* = .915
	MLCR	19%	19%	15%	46%	

**Figure 4.**
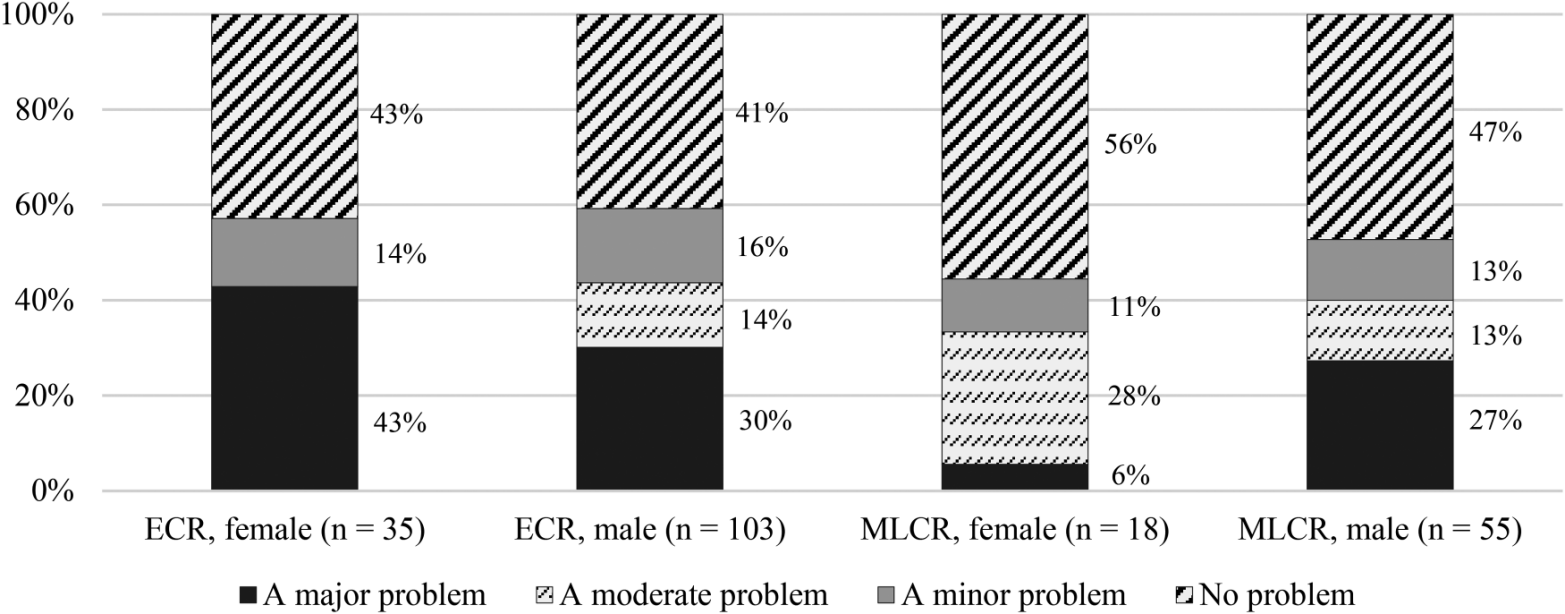
Severity ratings for the statement, ‘Collaborators insisting on co-authorship without having made contributions’, by research career stage and gender. *Note*. The Fisher-Freeman-Halton exact test revealed a statistically significant association between the intersectional variable (career stage by gender) and the rating of severity for the relevant item (test value = 16.456, *p* < .05). A pairwise Z-test with the critical p-value adjusted according to the Bonferroni method was used as a follow-up test.

 Figure 4 brings gender into the picture, showing the only item of self-interestedness that is significantly related to the gender-by-career stage variable. According to a follow-up pairwise test, significantly greater percentages of both female ECRs (43%) and male ECRs (30%) compared to female MLCRs (6%) rated ‘collaborators insisting on co-authorship without having made contributions’ as a major problem.

## Discussion

Generally speaking, all collaborative research should be guided by the same values of honesty, fairness, trustworthiness, and respect for others (Bozeman & Youtie, 2016; National Academy of Sciences, 2009). However, as has been found in the past, it is not unusual for researchers to fall short of these values and to engage in behaviours reflecting self-interestedness, including disregarding the views of others, claiming recognition on factors other than merit, and abusing research positions to take advantage of the order of authorship in publication (Bozeman et al., 1999). This study focused on six instances of self-interestedness among research collaborators as experienced by researchers in Zimbabwe, a developing country in southern Africa. It explored how the perceptions of the self-interestedness of collaborators differed according to the career stage and the nature of collaboration of the Zimbabwean researchers, and also considered gender as an intersectional variable.

Before reflecting on the results, the survey response rate of 13% warrants a few comments. Although low, the rate is consistent with that of similar online surveys of researchers on the African continent. For example, an online survey on the research practices and challenges of the next generation of scientists in Africa, conducted in 2016, recorded a response rate of 17% for Zimbabwe (Beaudry et al., 2018). The same survey also reported low response rates for five more countries, namely South Africa (12%), Ghana (11%), Nigeria (10%), Kenya (9%), and Egypt (4%). An online survey of research utilisation among researchers at three African universities also produced low response rates (Boshoff et al., 2018). Even though the online survey was combined with the administration of paper copies of questionnaires to increase participation, the final response rates were 22% for the University of Rwanda, 13% for the University of Ibadan in Nigeria, and 8% for the University of Nairobi in Kenya. These surveys, like the current one, illustrate the challenge of conducting surveys on ‘research about aspects of research’ in the African context. A response rate of 13% was therefore considered acceptable (although not ideal), and the present study should at most be interpreted as exploratory, requiring follow-up investigation, especially investigation of a qualitative nature. The questionnaire items on self-interestedness also require further development. What may be needed is conceptual reflection on the different dimensions of self-interestedness, with carefully worded items to be developed to map onto these dimensions.

The current study can therefore best be described as a pilot study that is worth refining and developing, with more respondents and more questions. Three sets of findings emerged from this ‘pilot’, each worthy of further investigation. The first concerns general observations of self-interestedness. Based on the results of the study, a temptation may exist to dismiss the challenge of self-interestedness in research collaboration in Zimbabwe as exaggerated, given that relatively large percentages of respondents (38% to 53%) considered five of the six items as either no problem in their collaboration, or not applicable to them. As a counterweight, however, it should be emphasised that notable percentages of respondents (at least 20%) considered each of the six items of self-interestedness a major problem in their own collaborations. With larger samples and more items, a comparison of the collaboration dynamics of the two opposite groups of severity (‘major problem’ vs. ‘no problem’) would provide useful insights.

Second, the study shows that Zimbabwean researchers involved exclusively in national collaboration report a greater degree of self-interestedness of collaborators than Zimbabwean researchers involved in international collaboration (with or without concomitant national collaboration). By implication, therefore, Zimbabwean research collaborators are more prone to self-interestedness than international research collaborators. This finding contradicts not only the stated hypothesis, but also existing narratives in the literature on unequal North-South partnerships, in which it is implied that researchers in the North ‘dominate’ those in the South (see studies by Boshoff, 2009; Gaillard, 1994; Okwaro & Geissler, 2015). One explanation for this ‘anomaly’ may be that international collaboration, as part of internationally funded projects, is guided by explicit guidelines on authorship and data sharing. In contrast, guidelines on data usage and authorship credit are not often specified in national collaborative engagements. Interactions between researchers involved in national collaboration are often informal (Sugimoto & Larivière, 2018), based on trust (real or imagined) and influenced by pre-existing personal or working relationships (Owusu-Nimo & Boshoff, 2017). It is in such informal collaborative arrangements that instances of undesirable collaborator behaviour are experienced (Bozeman & Youtie, 2016). Bringing gender into the relationship between the nature of collaboration and perceived self-interestedness showed that the significant differences found existed only between men. Greater severity of the specific items of self-interestedness was associated with men in national collaboration and not international collaboration, thus refuting the stated hypothesis. However, the sizes of the groups analysed (especially for female researchers) were too small to draw any firm conclusions about the interweaving of gender in said relationship.

Third, the study shows that there is no significant relationship between research career stage and perceptions of the severity of collaborator self-interestedness. This contrasts with the stated hypothesis, which is based on the assumption that junior researchers have negative collaborative experiences more often than senior researchers (Tsai et al., 2016; Youtie & Bozeman, 2016). A probable reason why experiences of collaborator self-interestedness do not differ according to research career stage could be related to the nature of collaboration. In general, instances of perceived self-interestedness were mostly a challenge for respondents involved in national collaboration. However, the profiling of researchers according to research career stage has shown that the ECR and MLCR groups do not differ significantly in terms of national collaboration. Specifically, 92% of respondents in both groups reported collaboration within their own organisation in Zimbabwe. Given this similarity in terms of intra-organisational collaboration, the absence of differences with regard to instances of collaborator self-interestedness between ECRs and MLCRs makes sense. Perhaps the real question should be why ECRs and MLCRs do not differ in terms of the extent of intra-organisational collaboration, and why such collaboration appears to be extremely high for both groups. Again, the answer may be obvious: the problem is the lack of international research and collaboration opportunities due to Zimbabwe's unique sociopolitical conditions.

Finally, the present study had limitations that must be acknowledged. As already mentioned, the response rate to the survey was not optimal. Thus, the data collected did not allow for more refined statistical analyses, such as regression analysis, to understand how the demographic and other variables together explain self-interestedness as an outcome. Another limitation is that the study relied solely on six statements in an online survey. Supplementary interviews would have provided more insight into the matter under review, and are therefore recommended for similar studies in other settings. Despite these limitations, however, it is believed that the study makes a significant contribution to the growing body of studies on research collaboration in the African context.

## Best Practice

As the study had a relatively low response rate, the findings of the study should be treated with caution. Measures aimed at promoting the norms of science in the African context, as well as research integrity for research organisations and research teams, are recommended here, provided that the evidence base for action is strengthened by follow-up studies. As a suggestion for the institutionalisation of best practice, organisations can invest in (i) the development of capacity-building programmes aimed at individual training, mentoring and awareness of ethical considerations related to the conduct of collaborative research, and in (ii) the development and regular updating of research collaboration policies, tools and guidelines to enable good collaborative practices. Individual research team members can be encouraged to attend training that establishes co-authorship best practices, including how to (i) make explicit decisions about co-authorship and data sharing before and during the collaboration process, (ii) speak up when treated unfairly within a team, and (iii) manage collaboration with colleagues who fail to uphold the values of trust, respect and integrity.

## Research Agenda

The current study could be replicated in one or more other developing countries in Africa. Zimbabwe could be argued to provide a special case, as the country has gone through a series of sociopolitical events that have affected its research system and its relationships with most research-funding organisations (Besada & Moyo, 2008). Many productive Zimbabwean researchers have also migrated to other countries, especially to neighbouring South Africa. Unlike other African countries, Zimbabwe is therefore likely to have more cases of researchers involved in national collaboration, as such collaboration would be a logical option for those researchers who have remained in Zimbabwe and who must create publication output in order to be promoted. On the other hand, as there is overwhelming evidence that African researchers in general collaborate more with researchers outside Africa than with researchers in Africa (Confraria et al., 2020; Guns & Wang, 2017), and considering the narratives about unequal North-South partnerships (Boshoff, 2009; Gaillard, 1994; Okwaro & Geissler, 2015), it may be that experiences of collaborator self-interestedness indeed occur predominantly in international collaborations. Therefore, it would be interesting to conduct a comparative study of the experiences of researchers in different categories of collaboration in at least one other African country that does not have politically strained relations with the international community.

## Educational Implications

Although some of the issues of self-interestedness in this study did not generally emerge as major issues of concern to large numbers of respondents, notable percentages of respondents nevertheless identified them as major concerns in their own collaborations. It is against this background that the study may have implications for the training of individual researchers and institutional research leaders and managers (i.e., those responsible for the formulation and implementation of research policy). One such implication is that research development programmes should include training on best collaboration skills and practices. Example could be skills in maintaining the values of trust, respect, integrity and appreciation during collaboration. Such values are central to establishing and maintaining collaborative research relationships (Bozeman et al., 1999). Moreover, although only one of the six statements of collaborator self-interestedness in this study reflected on publication co-authorship, the issue of co-authorship contribution was perceived as a major challenge mostly by researchers in national collaborations. Relevant programmes can therefore be considered to address issues of research ethics, and specifically credit-sharing procedures in co-authorship. Bozeman and Youtie (2016) warn that, in the absence of rules and procedures, credit disputes regarding co-authorship are likely to worsen.

## Conclusion

Not only the extent and severity of collaboration self-interestedness is worth investigating, but also the effects of self-interestedness on actual research outcomes, both through systematic research and by focusing on selected examples from practice. It is trusted that this exploratory study on collaborator self-interestedness as perceived by Zimbabwean researchers will stimulate more research activity in this area on the African context.
